# A Rabbi Speaks to a Medical Class

**Published:** 1877-07-20

**Authors:** 


A Rabbi Speaks to a Medical Class. Rosenspits, a Jewish Rabbi, made the closing address to the class of the Nashville Medical College, on the subject of their duties after graduation. His address was original and peculiar. He holds that the physician's duty involves the intellectual and moral not less than the physical well-being of his patients. In conclusion, he advised them to high aspirations, affirming that it was possible for all to obtain eminence in the profession, giving them as a motto the maxim of the Grecian philosopher, Themistocles —"If you *will* what you *can*, you can what you will.

